# Metformin-Loaded Fusogenic Liposome Improves the Therapeutic Efficacy and Safety of Doxorubicin in a Breast Cancer Treatment

**DOI:** 10.1021/acsomega.5c08220

**Published:** 2025-11-26

**Authors:** Thaís Mendes Pinheiro, Thaís Cristina de Amaral Almeida, Juliana de Oliveira Silva, Júlia Lobato Lopes, Geovanni Dantas Cassali, Marilia Martins Melo, Marthin Raboch Lempek, Raquel da Silva Ferreira, Danyelle M. Townsend, Elaine Amaral Leite, André Luis Branco de Barros

**Affiliations:** † Department of Pharmaceutical Products, Faculty of Pharmacy, 28114 Universidade Federal de Minas Gerais, Belo Horizonte, Minas Gerais 31270-901, Brazil; ‡ Department of General Pathology, Institute of Biological Sciences, Universidade Federal de Minas Gerais, Belo Horizonte, Minas Gerais 31270-901, Brazil; § Department of Veterinary Clinic and Surgery, Faculty of Veterinary Medicine, Universidade Federal de Minas Gerais, Belo Horizonte, Minas Gerais 31270-901, Brazil; ∥ Department of Drug Discovery and Biomedical Sciences, 2345 Medical University of South Carolina, Charleston, South Carolina 29425, United States; ⊥ Department of Clinical and Toxicological Analyses, Faculty of Pharmacy, Universidade Federal de Minas Gerais, Belo Horizonte, Minas Gerais 31270-901, Brazil

## Abstract

Breast cancer is a tumor with high incidence and mortality rates worldwide. Chemotherapeutic treatment consists of the systemic use of anticancer agents such as Doxorubicin (DOX). Recently, Metformin (MET), an antidiabetic drug, has been studied as an adjuvant in cancer treatment due to its action on proteins that regulate cell proliferation. DOX and MET have distinct drug distribution and pharmacokinetic parameters. Thus, strategies to equalize the delivery of these drugs to tumor tissue have been developed. In this context, liposomes are a promising alternative for increasing the effectiveness of cancer treatment with DOX and MET. This study aimed to prepare, characterize, and evaluate the antitumor activity of fusogenic liposomes containing DOX or MET. The liposomes were prepared by the Bangham method and characterized physicochemically. The prepared nanosystems (Lip-MET and Lip-DOX) showed diameters of approximately 120 nm, polydispersity index lower than 0.3, zeta potential close to neutrality, and drug encapsulation content of 98.8% ± 18.7 for Lip-DOX and 10.1% ± 0.5 for Lip-MET. To evaluate the antitumor activity, 4T1 breast tumor-bearing mice were used as a model. Once the tumor reached ∼100 mm^3^, mice received four administrations (on days 1, 3, 5, and 7), each containing 5 mg/kg of DOX and 15 mg/kg of MET. A significant decrease in tumor volume was observed in animals treated with Lip-DOX + Lip-MET, compared to the other groups, evidenced by a tumor growth inhibition rate of 87.2%. It is also noteworthy that the Lip-DOX + Lip-MET treatment resulted in a significant decrease in lung and liver metastases. In these animals, 1–3 foci of lung metastases were observed, compared to control animals that reached 7–10 foci. In addition, 100% of the animals treated with free DOX presented arrhythmias, while only 40% of the animals treated with Lip-DOX + Lip-MET presented these cardiac alterations. Therefore, the coadministration of liposomes loading DOX and MET showed promise for increasing antitumor activity and safety in breast cancer treatment.

## Graphical abstract

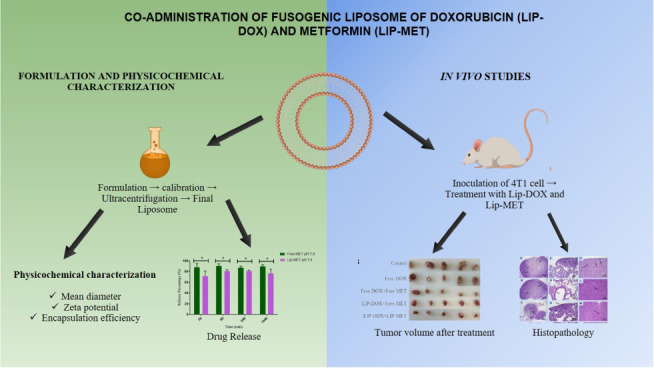

## 1 Introduction

Breast cancer is a global health challenge and remains a significant public health concern. It is estimated that by 2045, there will be 3.36 million new cases of breast cancer and 1.06 million deaths worldwide.^1–3^ Significant advances in the pharmacological treatment of cancer have been made in recent years, but there are still challenges. Among the drugs used, doxorubicin (DOX) stands out. DOX is an antibiotic of the anthracycline class, widely used for breast cancer.^4^ The main DOX mechanisms of action are the inhibition of type II topoisomerase and the intercalation of DNA chains that alter the synthesis and structure of the nucleic acids, affecting cell replication and apoptosis.^5,6^

Despite its worldwide use and high efficiency against tumor cells, DOX treatment presents severe toxic effects, such as cardiotoxicity. DOX penetrates cardiac tissue cells, increasing sarcoplasmic calcium and generating changes in the electrocardiogram (ECG). The most commonly observed electrophysiological changes are prolongation of the QT and QRS intervals and defects in cardiac electrical conduction, which result in arrhythmias and often limit chemotherapy treatment.^7,8^ Considering these challenges, new strategies are needed to control the progression of cancer and reduce the toxicity of the drugs traditionally used. It is well-known that combining two or more chemotherapeutic agents is a viable approach to improving the efficacy of antitumor treatment and increasing the likelihood of achieving a cure for the patient compared with monotherapy. The combination with metformin (MET) has been suggested due to its potential antitumor activity. MET is a biguanide hypoglycemic drug used in type II diabetes treatment and acts on the bioenergy pathways of cells. It has been studied in cancer therapy by reducing the production of AMP-activated protein kinase (AMPK) and turning off the adenosine triphosphate (ATP) consumption pathways. This activation inhibits the mTOR protein, essential for cell growth, which leads to a reduction in the rate of cell proliferation, especially in tumor cells. In addition, MET exhibits several anticancer effects, including the reduction of cell proliferation, induction of cell cycle arrest, and activation of programmed cell death mechanisms such as apoptosis and/or autophagy. Recent evidence also suggests its ability to trigger alternative forms of cell death, such as pyroptosis, an inflammatory and caspase-dependent process. Furthermore, studies indicate that MET decreases cell motility and invasiveness while enhancing cell adhesion in various solid tumor models.^9^ An additional effect of MET is the ability to reduce calcium concentrations in the sarcoplasmic reticulum. Its utility in reducing DOX-induced cardiotoxicity has been studied.^5,10,11^

Off-target effects of systemic delivery of drugs are a problem. Nanoencapsulation has proven to be highly effective in reducing toxic effects and enhancing the antitumor efficacy of drugs used in breast cancer treatment by increasing targeting to tumor cells while minimizing impact on healthy cells. Liposomes have emerged as a strategy for delivering drugs to the tumor microenvironment while potentially reducing systemic toxicity. They are nanosystems highly biocompatible and capable of encapsulating numerous hydro- and lipophilic molecules. Liposomes are taken up by cells and release their contents into the cytoplasm, allowing them to reach the site of action. This process can be facilitated by fusion with cell membranes, particularly through the use of fusogenic liposomes.^12–14^ These properties are due to the lipid compounds of the liposome, dioleoylphosphatidylethanolamine (DOPE), a structural membrane lipid that requires a stabilizing agent, cholesteryl hemisuccinate (CHEMS). Furthermore, the addition of distearoylphosphatidylethanolamine-*N*-(polyethylene glycol)­2000 (DSPE-PEG2000), a lipid with polyethylene glycol (PEG) chains, contributes to reduce aggregation between vesicles, increase the shelf stability of the formulation, and reduce recognition by the Mononuclear Phagocytic System cells (MPS), increasing circulation time in the body.^13,14^

Thus, the combination of MET and DOX encapsulated in liposomes represents a promising therapeutic strategy for breast cancer treatment. Studies have shown that the release of MET in the tumor microenvironment exerts antitumor effects by reducing hypoxia, HIF-1α, and P-glycoprotein expression. These changes significantly enhance DOX cytotoxicity in vitro and lead to tumor regression in vivo.^15^ Additionally, in triple-negative breast cancer models, a metronomic regimen using pegylated liposomal DOX in combination with MET reduced cancer stem cell markers and inhibited the Wnt/β-catenin pathway, resulting in a strong antitumor response.^16^ Collectively, these findings support the synergistic potential of liposomal delivery of DOX and MET, highlighting targeted drug delivery and modulation of the tumor microenvironment as key factors for highly effective breast cancer therapy.^17^ Therefore, this study aimed to develop liposomal formulations containing DOX (Lip-DOX) and MET (Lip-MET) that, when coadministered, could increase antitumor activity. To achieve this, 4T1 breast tumor-bearing BALB/c mice were used as the experimental model. In addition, toxicological parameters were evaluated through histopathological and electrocardiographic analyses.

## 2 Materials and Methods

### 2.1 Materials

Doxorubicin hydrochloride (DOX) was donated by Eurofarma (Säo Paulo, Brazil). Metformin (MET) was purchased from Bs Pharma (Belo Horizonte, Brazil). DOPE and DSPE-PEG2000 were purchased from Lipoid GmbH (Ludwigshafen, Germany). Polycarbonate membranes were purchased from Millipore (Billerica, USA). CHEMS was purchased from Sigma-Aldrich (Säo Paulo, Brazil). Dubelcco’s Modified Eagle’s Medium (DMEM) was purchased from Sigma-Aldrich (Säo Paulo, Brazil). PSA antibiotics (penicillin, streptomycin, and amphotericin B) and trypsin were purchased from Invitrogen (Thermo Fisher Scientific—Säo Paulo, Brazil). Fetal bovine serum (FBS) was purchased from Gibco (Säo Paulo, Brazil). Xylazine solution (Dopaser 2%) and ketamine hydrochloride solution (Dopalen 10%) were purchased from Ceva Brasil (Paulínia, Brazil). All other reagents and chemicals were purchased at analytical grade.

### 2.2 Methods

#### 2.2.1 Liposome Preparation

Liposomes were prepared using the lipid film hydration technique,^18^ followed by size calibration. First, aliquots of DOPE, CHEMS, and DSPE-PEG2000 lipids dissolved in chloroform (5.8:3.7:0.5 molar ratio, respectively; total lipid concentration 20 mM) were transferred to a round-bottom flask. The solvent was removed at low pressure to prepare a thin lipid film. Afterward, an aliquot of 0.1 M NaOH solution was added to the flask to promote complete ionization of the CHEMS molecules, and then the lipid film was hydrated with 300 mM aqueous ammonium sulfate solution at room temperature, under vigorous stirring.^19^ The obtained liposomes were calibrated by extrusion with 0.2 and 0.1 μm polycarbonate membranes, in 5 cycles for each membrane, using a Lipex Biomembranes extruder, Model T001 (Vancouver, Canada). Subsequently, the liposome suspension was subjected to ultracentrifugation (Ultracentrifuge Optima L-80XP, Beckman Coulter, Brea, USA) at 150,000*g*, 4 °C, for 120 min, for purification and removing unencapsulated ammonium sulfate. The pellets were resuspended with HEPES buffer pH 7.4 to obtain blank liposomes (Lip-Blank). For Lip-DOX, DOX powder (2 mg/mL) was added to Lip-Blank dispersion, and after complete solubilization, it was incubated overnight (∼16 h) at 4 °C to promote drug encapsulation by the ammonium sulfate gradient method. The ammonium sulfate gradient method enables high DOX encapsulation by driving the drug into liposomes through a transmembrane ion gradient, where it forms stable DOX–sulfate complexes inside the vesicles. The final purification is not necessary due to the high encapsulation rate of the DOX.^20,21^

The preparation of Lip-MET followed a procedure similar to that described for Lip-DOX, with the main difference being the step in which the drug was added. MET was dissolved in ammonium sulfate during the film hydration at a concentration of 50 mg/mL. The unencapsulated drug was then removed by ultracentrifugation and resuspended as previously described.^19^

#### 2.2.2 Liposome Physicochemical Characterization

##### 2.2.2.1 Mean Diameter, Polydispersity Index (PDI), and Zeta Potential

The mean diameter and polydispersity index (PDI) of Lip-DOX and Lip-MET were determined by dynamic light scattering (DLS) at 25 °C at a 90° angle, as previously described.^19^ The zeta potential was determined by electrophoretic mobility in combination with DLS. Both parameters were measured using the Zetasizer NanoZS90 equipment (Malvern Instruments, Worcestershire, UK). All samples were diluted in HEPES buffer pH 7.4 at a ratio of 1:100 and measured in triplicate.

##### 2.2.2.2 Determination of DOX and MET Encapsulation Percentage

DOX quantification was performed by spectrophotometry (UV–vis) using Thermo Scientific Evolution 201 equipment (Madison, USA) connected to the Thermo INSIGHT software. Methanol:water (40:60 v/v) solution was used to dissolve the samples and the wavelength of 480 nm was selected for readings. To determine DOX in Lip-DOX, the lipid membrane was opened in isopropyl alcohol in a volumetric ratio of 1:10 (liposomes:isopropyl alcohol) before dilution. The DOX concentration was determined before (total DOX) and after (DOX ultrafiltered) centrifugation at 12,000*g* for 20 min using ultrafiltration tubes (Amicon, 30 kDa, Danvers, USA).^19,21^ The encapsulation percentage (EP %) was calculated according to the following eq 1:

1 %EP=100−(DOXultrafiltered/totalDOX)×100

A similar method was used for the quantification of MET. The absorbance was read at 237 nm, and a solution composed of Lip-Blank:isopropyl alcohol:methanol (1:2:7 volumetric ratio) was used as a reading blank. Afterward, the Lip-MET samples were opened with isopropyl alcohol, in a volumetric ratio of 1:10 (liposomes: isopropyl alcohol) and then diluted in methanol:water solution (40:60). The MET concentration was determined before (total MET) and after ultracentrifugation (MET encapsulated) at 50.000 rpm for 90 min, and the encapsulation percentage (EP %) was calculated according to the following eq 2:

2 %EP=(METencapsulated/totalMET)×100

#### 2.2.3 In Vitro Release MET

The release profile of MET from Lip-MET was determined by the dialysis method. Dialysis membranes (cellulose ester membrane, MWCO 14 kDa; Sigma-Aldrich, St Louis, USA) were filled with 500 μL of MET solution (5 mg/mL) or Lip-MET, sealed, and incubated with 50 mL of HEPES buffer pH 7.4 for 24 h at 37 °C, under orbital stirring at 150 rpm. At the time points of 30 min, 60 min, 240 min, 1440 min, 500 μL of external phase were removed, and the volume of the acceptor phase was replaced. The MET concentration was analyzed by the UV method described above. Values were plotted as cumulative percentages of drug release.

#### 2.2.4 Stability Lip-MET

Lip-MET were prepared (*n* = 3) and stored in the refrigerator at 4 °C to evaluate storage stability. Immediately after preparation and on days 1, 3, 7, 15, 30, and 60, aliquots were withdrawn and the % EP, mean diameter, zeta potential, and PDI were evaluated as previously described.

#### 2.2.5 In Vivo Studies

##### 2.2.5.1 Cell Culture and Animals

Murine breast carcinoma cells (4T1) were cultured in RPMI1640 medium supplemented with 10% (v/v) FBS and 1% PSA (penicillin, streptomycin and amphotericin). The cells were maintained at 37 °C and 5% CO2 in a humidified atmosphere.

Female BALB/c mice aged 7–8 weeks were obtained from the “Centro de Bioterismo da Universidade Federal de Minas Gerais (CEBIO/UFMG)”. The animals were kept in a light and temperature-controlled environment with free access to water and food. All animal studies were approved by the Ethics Committee on the Use of Animals (CEUA/UFMG) under protocol number 135/2023.

##### 2.2.5.2 Antitumor Activity

A suspension containing 1 × 10^6^ 4T1 murine breast tumor cells was inoculated into the right flank of the animals. The tumor growth was monitored, and once the tumor volume reached approximately 100 mm^3^ (∼12 days), the animals were randomly divided into five groups (*n* = 7). These groups received intravenously: Saline 0.9% (w/v) (Control Group); free-DOX; free-DOX + free-MET; Lip-DOX + free-MET; Lip-DOX + Lip-MET. The animals received four doses of 5 mg/kg of DOX and 15 mg/kg of MET every 2 days, reaching a cumulative dose of 20 mg/kg of DOX and 60 mg/kg of MET (Figure 1). The tumor volume (TV) and body weight were measured at the same time point. The TV (mm^3^) was calculated by eq 3:

3 $$\mathrm{TV}={(d_{1})}^{2}\times d_{2}\times 0.5$$

where *d*_1_ and *d*_2_ represent the shortest and longest diameters, respectively.

**Figure 1:**
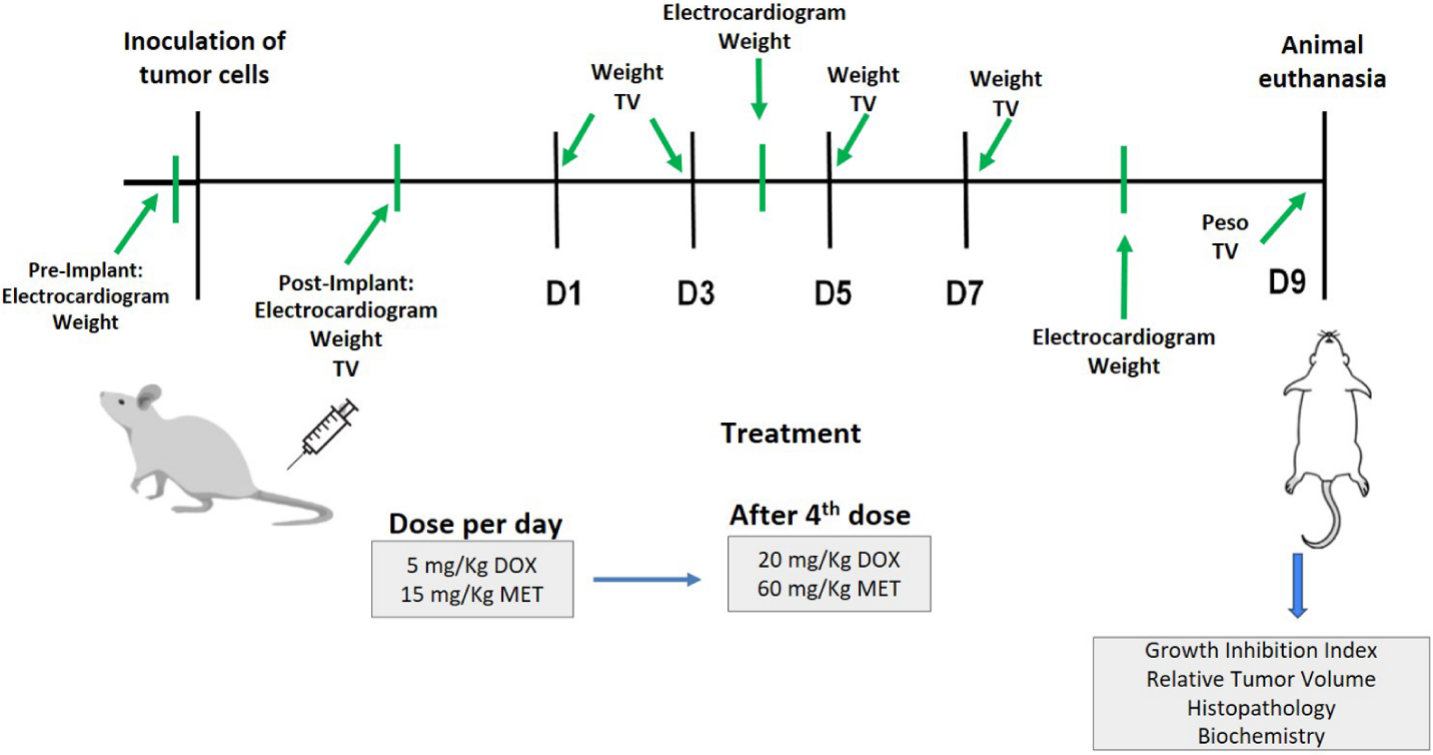
Flowchart representing the steps of the antitumor efficacy study in 4T1 tumor-bearing mice. Tumor efficacy study. Tumor Volume (TV); Doxorubicin (DOX); Metformin (MET); D1: Day 1 of treatment; D3: Day 3 of treatment; D5: Day 5 of treatment; D7: Day 7 of treatment.

At the end of the experimental period, the relative tumor volume (RTV) was calculated, using eq 4,^20,22^ to estimate the ratio of increase or reduction in volume according to the initial volume. (D9) euthanasia day; (D1) first day of treatment.

4 RTV=TumorvolumeD9/TumorvolumeD1

The percentage of tumor growth inhibition rate (IR) was also determined using eq 5:^23^

5 IR=(RTVtreatedgroup/RTVcontrolgroup)×100

The animals were weighed before the start of treatment and before each dose was administered. The result was calculated based on the percentage of weight loss compared to the beginning of treatment. Finally, the animals were anesthetized with ketamine and xylazine (80 mg/kg and 15 mg/kg, respectively) and then euthanized. The organs (heart, liver, lung, kidneys, and tumor tissue) were removed for histopathological analysis.

##### 2.2.5.3 Histopathological Analysis

The collected organs were fixed in 10% formalin for 48 h, then dehydrated with alcohol, embedded in paraffin blocks, sectioned into 4 μm-thick sections and stained with hematoxylin and eosin. The images were evaluated using an Olympus BX-40 optical microscope (Olympus, Tokyo, Japan). The number of lung metastases was counted individually and expressed as a score: score: 0, no metastases detected; +, 1–3 metastatic foci; ++, 4–7 metastatic foci; +++, 8–10 metastatic foci; ++++, >10 metastatic foci.^19,24^

##### 2.2.5.4 Electrocardiographic Analysis

The ECG analyses of the animals were acquired before tumor cell implant, before treatment, after the second treatment dose, and before euthanasia as indicated in Figure 1. The ECG was noninvasively acquired using a veterinary electrocardiograph system (InCardio—Inpulse Animal Health, Florianopolis, Brazil). The animals were manually restrained in dorsal decubitus, and the ECG and heart rate (HR) were monitored continuously for 02 min and mainly analyzed in the DII derivation. Subsequently, registers in 120 s windows were selected to analyze 5 to 9 complete cardiac cycles. The recordings consisted of measurements of the QT and QTc interval (interval between the beginning of a Q wave and the end of a T wave on the ECG), PR interval (interval between the beginning of a P wave and the end of an R wave), QRS complex (includes three waves: Q, R, and S, measuring from the beginning of the Q wave to the end of the S wave), T wave, P wave (analysis of atrial activation), and HR (heart rate of the animals). The QTc values were calculated with the Fridericia formula: QTc = QT/^3^√RR. The tracings were also evaluated for arrhythmias and other morphological alterations.

##### 2.2.5.5 Statistical Analysis

Data were expressed as mean ± standard deviation of mean (SD). The D’Agostino and Pearson and Brown–Forsythe tests were used to verify the normality and homoscedasticity. The variables that did not present normal distribution were transformed, when appropriate, by equation *y* = log­(variable). Data were tested by ANOVA followed by a Tukey posttest. Differences were considered significant when *p*-values were <0.05, with a 95% confidence interval. All data were analyzed by GraphPad PRISM software, version 9.00 (GraphPad Software Inc.).

## 3 Results

### 3.1 Physicochemical Characterization of Liposomes

The physicochemical properties of Lip-DOX and Lip-MET are shown in Table 1. Both liposome formulations showed a mean diameter within the range of 100–150 nm. Besides, the formulations are monodisperse with PDI lower than 0.3. The zeta potential for both formulations was close to neutrality due to PEG chains on the liposome surface.

**Table 1:** Physicochemical Characteristics (Mean Diameter, PDI, Zeta Potential, and EP) for the Different Liposomal Formulations Containing the Encapsulated Drugs^a^

	Lip-Blank	Lip-DOX	Lip-MET
Mean diameter (nm)	130.2 ± 19.8	108.0 ± 2.0	129.1 ± 4.3
PDI	0.14 ± 0.04	0.09 ± 0.3	0.09 ± 0.01
Zeta Potential (mV)	–8.9 ± 4.3	–2.4 ± 0.6	–3.1 ± 0.8
EP (%)	-	99.5 ± 0.3	10.2 ± 0.5
Drug loading (mg/mL)	-	2.0 ± 0.3	5.0 ± 0.5

a Results are expressed as the mean ± SD (*n* = 3). PDI means Polydispersity Index and EP means Encapsulation Percentage.

The EP of Lip-DOX showed values close to 100%. This high efficiency is attributed to the complex formation between doxorubicin hydrochloride and ammonium sulfate, which induces DOX precipitation in the liposome core, thereby enhancing the encapsulation rate.^25,26^

### 3.2 Release Lip-MET

Figure 2 shows the release profiles for free-MET and Lip-MET. Free-MET had a significantly faster release profile than Lip-MET.

**Figure 2:**
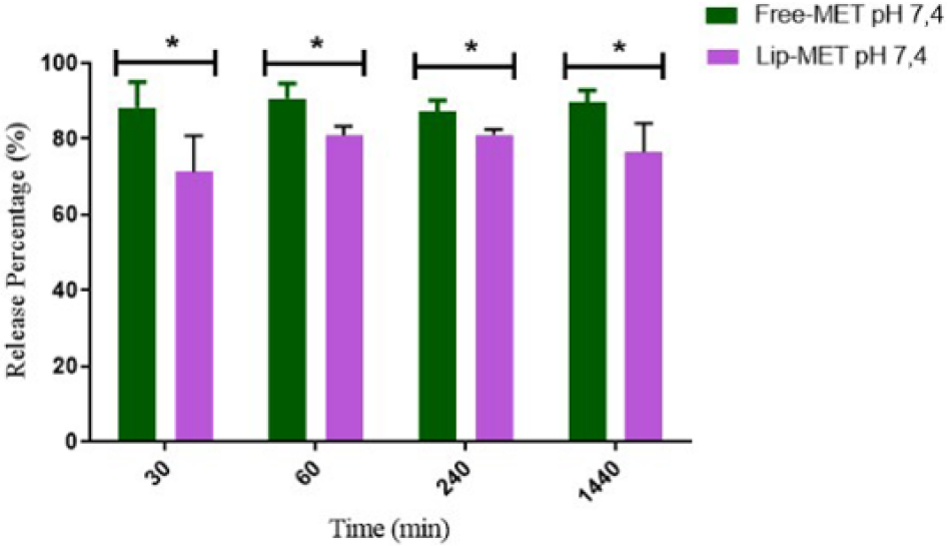
Release profile of Lip-MET and Free-MET at pH 7.4 at different times. Study of Lip-MET and free-MET release in function of time (min). Results are expressed as the mean ± SD (*n* = 5). All data were analyzed by one-way ANOVA analysis of variance. *Represents a significant difference between Lip-MET compared to free-MET (*p* < 0.05).

A significant difference between the MET liposome and free-MET was observed at all time points. The liposomes, as demonstrated, showed a controlled release of MET, obtaining values lower than those achieved by the free drug throughout the study (Figure 2). The release of Lip-MET reaches approximately 70% in 30 min and after 60 min, it reaches approximately 80%, which remains until 24 h. On the other hand, free-MET released was around 90% at all time points. These results indicated that liposomes could control the release of the MET, especially in the initial times, providing reassurance about its potential use in drug delivery systems.

### 3.3 Stability

In the present study, the stability of Lip-MET under storage at 4 °C was evaluated by measuring parameters such as mean diameter, PDI, zeta potential, and % EP up to 60 days. Table 2 showed that Lip-MET was stable for 30 days, with a significant increase in mean diameter and PDI observed on day 60. Regarding % EP, a reduction of approximately 20% was observed after day 1, probably due to the release of the adsorbed drug on the liposome surface. However, the concentration remained stable at around 70% of the initially encapsulated MET.

**Table 2:** Stability Study of Lip-MET under Storage at 4 °C, Zeta Potential, Mean Diameter, PDI, and Encapsulation Efficiency (EP) Were Evaluated for Up to 60 Days^a^

	Mean diameter (mn)	PDI	Zeta potential (mV)	EP (%)
Day 0	120.5 ± 2.1	0.10 ± 0.01	–5.6 ± 0.3	10.5 ± 0.1
Day 1	135.9 ± 3.9	0.09 ± 0.05	–4.0 ± 0.2	8.6 ± 4.4
Day 3	138.4 ± 2.2	0.12 ± 0.01	–4.1 ± 0.2	7.9 ± 3.5
Day 7	131.8 ± 1.9	0.10 ± 0.01	–4.9 ± 0.1	7.6 ± 4.4
Day 14	138.2 ± 5.0	0.13 ± 0.01	–5.1 ± 0.6	8.1 ± 2.6
Day 30	133.7 ± 1.0	0.12 ± 0.04	–5.9 ± 0.9	7.2 ± 2.2
Day 60	176.2 ± 35.6*	0.36 ± 0.07*	–3.5 ± 1.2	8.1 ± 4.6

a Results are expressed as the mean ± SD (*n* = 3). All data were analyzed by one-way ANOVA analysis of variance followed by Tukey’s posttest. * Significant difference compared to other days (*p* < 0.05). PDI means Polydispersity Index and EP means Encapsulation Percentage.

### 3.4 Antitumor Activity

The antitumor activity was evaluated in vivo using BALB/c mice bearing 4T1 breast tumors. Significant differences in tumor growth were observed between the control group and the other treatments: free-DOX, free-DOX + free-MET, Lip-DOX + free-MET, and Lip-DOX + Lip-MET (Figure 3). A significant difference was observed between the tumor volume of animals receiving free-DOX and those in combination with MET. The group treated with Lip-DOX + Lip-MET showed the lowest tumor growth rate throughout the experiment. These results show that the drug combination, as well as the nanoencapsulation (Lip-DOX/Lip-MET), was more effective in inhibiting tumor growth control when compared to the control or free-DOX-treated groups. These results were confirmed by RTV and IR (Table 3). It is important to highlight that the group treated with the liposome combination (Lip-DOX + Lip-MET) presented an RTV value at around 5-fold and 8-fold lower than the free-DOX and control group, respectively. These findings were reproduced in the IR values. Moreover, body weight of the mice was monitored throughout the treatment protocol (Figure 3C). Body weight analysis is a preliminary indicator of potential toxicity following drug administration and is commonly reported in doxorubicin regimens.^19^ In our study, we observed a reduction in body weight across all drug-treated groups. This finding reinforces the importance of conducting additional toxicity assessments, such as histopathological analysis of target organs.

**Figure 3:**
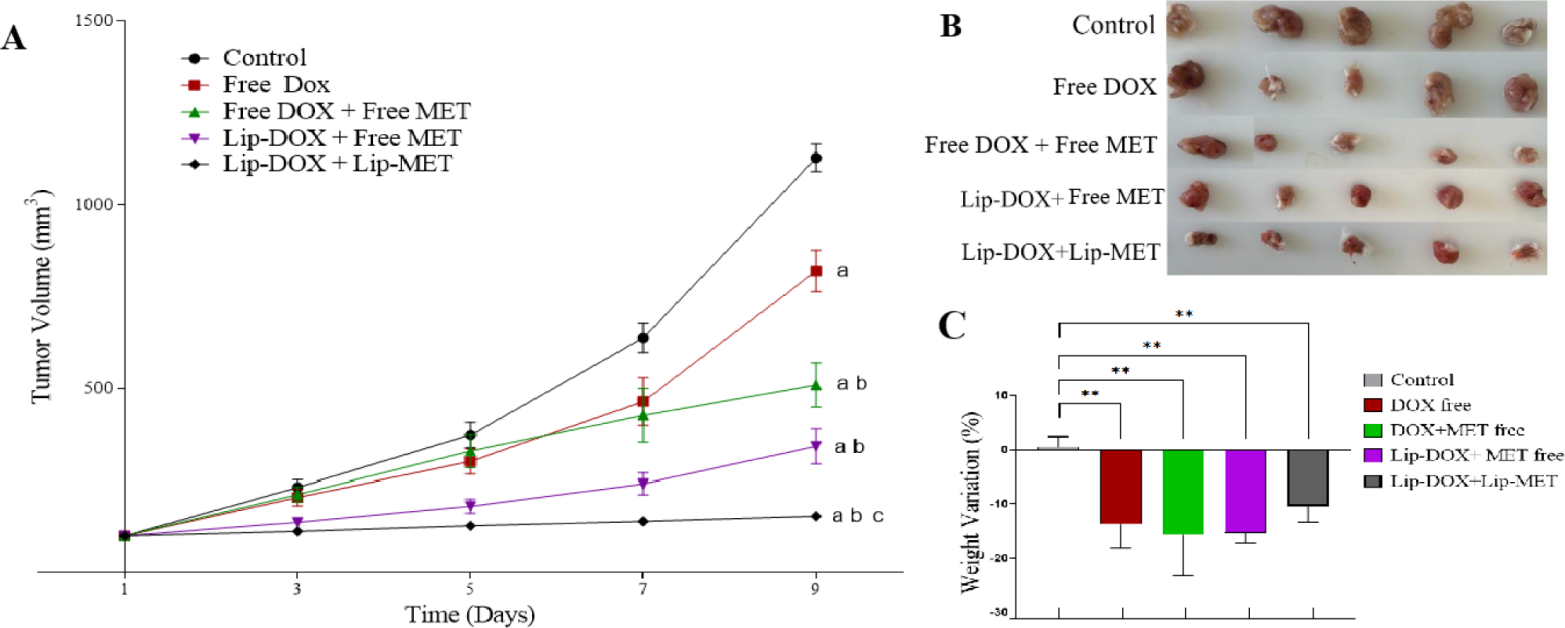
(A) Curve of tumor growth (mm^3^) for control and drug-treated mice, (B) Photographs of tumors after dissection of control and drug-treated animals, and (C) Percentage variation of body weight. Results are expressed as the mean ± SD (*n* = 7). All data were analyzed by one-way ANOVA analysis of variance followed by Tukey’s posttest. ^a^represents a significant difference compared with control group (*p* < 0.001), ^b^represents a significant difference compared with free DOX group (free DOX + free MET and Lip-DOX + free MET groups, *p* < 0.05; Lip-DOX + Lip-MET group, *p* < 0.001), and ^c^represents a significant difference compared with free DOX + free MET and Lip-DOX + free MET group (*p* < 0.001).

**Table 3:** Relative Tumor Volume (RTV) and Growth Inhibition Rate (IR) for Each Treatment^a^

Treatment	RTV	IR (%)
Controle	11.3 ± 0.8	-
Free-DOX	7.8 ± 2.6^b^	25.1
Free-DOX + free-MET	5.1 ± 1.3^b^ ^c^	54.9
Lip-DOX + free-MET	3.6 ± 1.3^b^ ^c^	67.9
Lip-DOX + Lip-MET	1.4 ± 0.2^b^ ^c^	87.2

a Results are expressed as the mean ± SD (*n* = 7). All data were analyzed by one-way ANOVA analysis of variance followed by Tukey’s post test.

b Represents a significant difference compared to the control (*p* < 0.05).

c Represents a significant difference compared to free-DOX (*p* < 0.05).

### 3.5 Histopathological Analysis

The tumors and organs were analyzed to detect necrosis, metastasis, and signs of toxicity. The histological analysis of the tumor is shown in Figure 4A–O. The neoplastic cells exhibited a round or oval shape, with a broad and eosinophilic cytoplasm characteristic of epithelial cells. A marked nuclear polymorphism was observed, with multiple large and prominent nuclei. Additionally, the cells were arranged in a solid pattern, typical of 4T1 tumors.^27,28^

**Figure 4:**
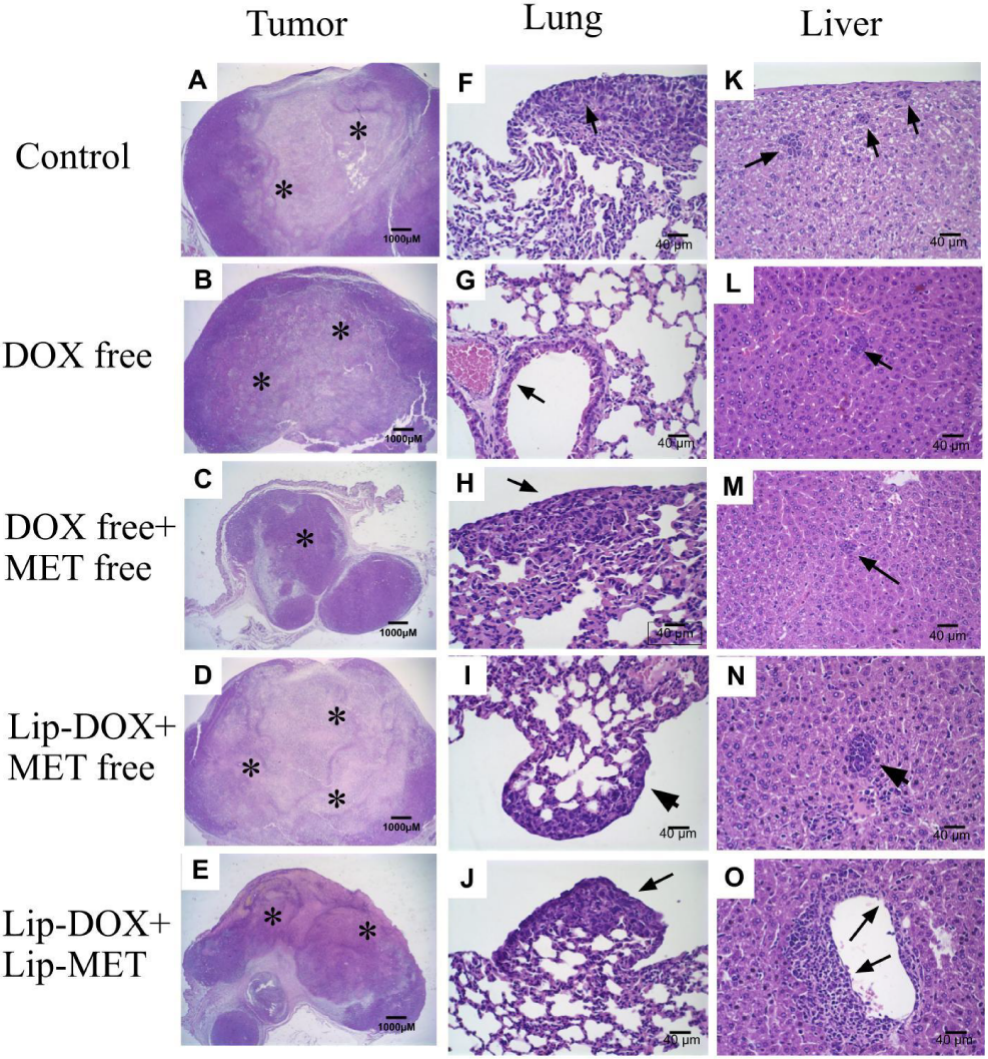
Histological sections of tumors from female BALB/c mice with 4T1 breast tumors in (A) control group; (B) free-DOX; (C) free-DOX + free-MET; (D) Lip-DOX + free-MET; (E) Lip-DOX + Lip-MET. Histological sections of lungs from female BALB/c mice with 4T1 breast tumors in (F) control group; (G) free-DOX; (H) free-DOX + free-MET; (I) Lip-DOX + free-MET; (J) Lip-DOX + Lip-MET. Histological sections of livers from female BALB/c mice with 4T1 breast tumors in (K) control group; (L) free-DOX; (M) free-DOX + free-MET; (N) Lip-DOX + free-MET; (O) Lip-DOX + Lip-MET. (A–E) magnification 2×; (F–O) magnification 40×. Black arrows indicate areas of metastasis. Asterisks indicate areas of necrosis.

The photomicrography of tumors treated with free-DOX, free-DOX + free-MET, Lip-DOX + free-MET, and Lip-DOX + Lip-MET showed an area of necrotic cells (Figure 4B–E, respectively). Notably, the group treated with Lip-DOX + Lip-MET exhibited a more extensive area of necrosis when compared to the other treated groups (Figure 4E). In addition, only the group that received Lip-DOX + Lip-MET presented skin ulceration in the tumor area.

4T1 breast tumors are aggressive and metastasize rapidly to other organs, especially to the liver and lungs.^29^ Therefore, these organs were analyzed for metastases (Figure 4). In the liver (Figure 4K–O), multifocal metastases with considerable inflammatory infiltrate were observed in the control group, but in all treatment groups, there was a significant reduction in the number of metastases, and only focal metastases were observed. Additionally, the liver photomicrographs showed perivascular degeneration of hepatocytes, characterized by vacuolated cells and discrete alteration of the usual tissue architecture. In the lungs analysis (Figure 4F–J), a significant number of metastases and intense perivascular inflammatory infiltrate were observed in 80% of the mice in the control group. As observed in the liver, the groups treated with free-DOX, free-DOX + free-MET, Lip-DOX + free-MET, and Lip-DOX + Lip-MET presented a drastic reduction in metastasis number and discrete perivascular inflammatory infiltrate.

Animals treated with free-DOX + free-MET and Lip-DOX + Lip-MET presented 1 to 3 foci in 20% of the animals, while 40% of the animals presented 1 to 3 foci of metastases in the group treated with free Lip-DOX + free-MET (Table 4). However, animals treated with free-DOX did not present metastasis foci in the lungs despite perivascular inflammatory infiltrate, as in the other groups (Figure 4G).

**Table 4:** Lung Metastasis Foci of 4T1 Tumor in Untreated (Control) and Treated (Free-DOX; Free-Dox + Free-MET; Lip-DOX + Free-MET; Lip-DOX + Lip-MET) Female BALB/c Mice^a^

		Control	Free-DOX	Free-DOX + free-MET	Lip-DOX + free-MET	Lip-DOX + Lip-MET
Score	Animal 1	+	0	0	0	0
	Animal 2	+++	0	0	0	+
	Animal 3	+	0	0	+	0
	Animal 4	++	0	+	+	0
	Animal 5	0	0	0	0	0

a Results are expressed by score: 0, no metastasis detected; + , 1–3 foci of metastasis; ++, 4–6 foci of metastasis; +++, 7–10 foci of metastasis in the lung.

The study also included a histological analysis of the kidneys and heart to verify potential toxicities. All groups (control and treated groups) showed preserved renal tissue with typical architecture. However, concerning cardiac toxicity, it is known that DOX promotes dose-dependent cardiotoxicity.^30,31^ It was possible to observe that all animals treated with DOX (free or liposomal form) showed areas of hyaline degeneration (Table 5), characterized by hypertrophic, eosinophilic, and vacuolated cardiac fibers in the left ventricle (Figure 5).

**Figure 5:**
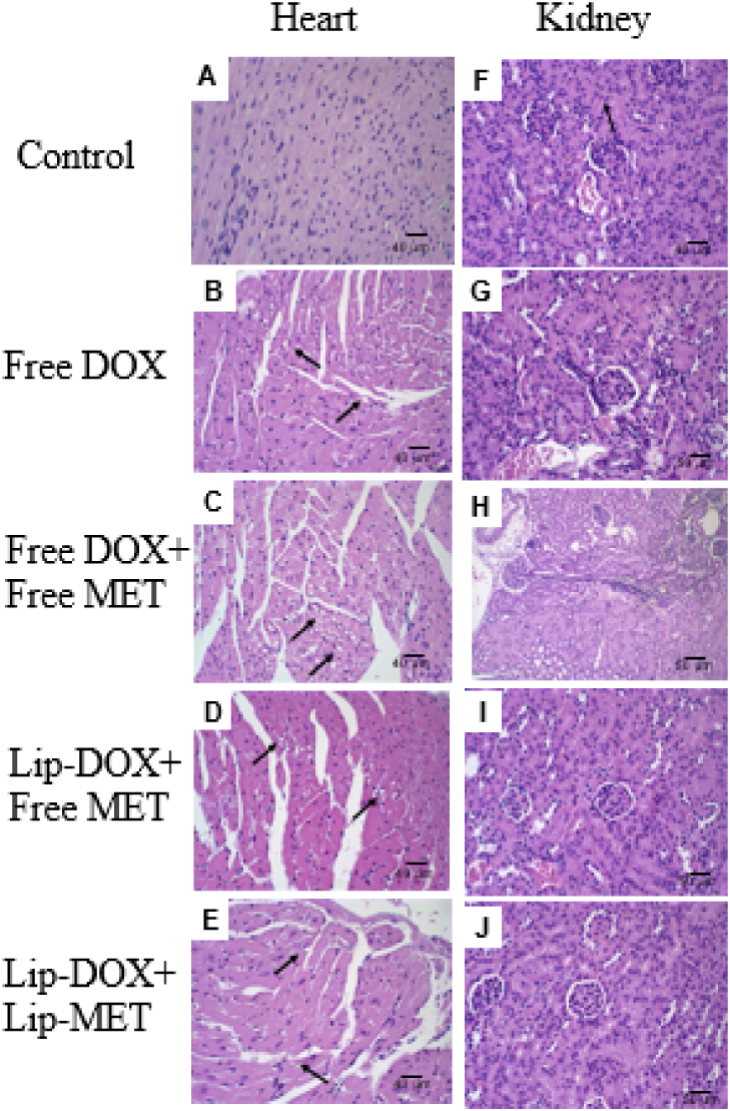
Histological sections of the heart of female BALB/c mice carrying 4T1 breast tumor in the (A) control group; (B) free-DOX; (C) free-DOX + free-MET; (D) Lip-DOX + free-MET; (E) Lip-DOX + Lip-MET. And kidney (F) control group; (G) free-DOX; (H) free-DOX + free-MET; (I) Lip-DOX + free-MET; (J) Lip-DOX + Lip-MET. Black arrows indicate areas of hyaline degeneration. Heart magnification 40×. Kidney magnification 20×.

**Table 5:** Description of the Extension and Intensity of Cardiac Hyaline Degeneration in Animals with 4T1 Breast Tumors Treated with the Formulations of Saline (Control), Free-DOX, Free-Dox + Free-MET, Lip-Dox + Free-MET, and Lip-Dox + Lip-MET

	Hyaline Degeneration	
	Extension	Intensity
Control	-	-
Free-DOX	Focal	Discrete
Free-DOX + free-MET	Focal	Moderate
Lip-DOX + free-MET	Focal	Discrete
Lip-DOX + Lip-MET	Focal	Discrete

### 3.6 Electrocardiographic Analysis

Cardiac arrhythmia events are shown in Table 6. The presence of arrhythmia, mainly characterized by ventricular or atrial extrasystoles, in the electrocardiographic (ECG) of all DOX-treated groups was expected, however, it was attenuated in the group treated with Lip-DOX + free-MET. This cardiotoxicity signal was observed in only two animals in the Lip-DOX + Lip-MET. However, significant prolongations in QT, QTc, QRS, and PR intervals were not observed in treated animals in this applied therapeutic scheme (data not shown).

**Table 6:** Electrocardiographic Changes at the End of Treatment of Female Balb/C Mice Carrying 4T1 Murine Breast Tumor^a^

	Control	Free-DOX	Free-DOX + free-MET	Lip-DOX + free-MET	Lip-DOX + Lip-MET
Animal 1	0	0	+	+	0
Animal 2	0	+	+	+	0
Animal 3	0	0	+	+	+
Animal 4	0	+	+	+	+
Animal 5	0	+	+	+	0

a Results are expressed by score: 0, no changes; +, ventricular and/or atrial extrasystole (arrhythmia).

## 4 Discussion

In this study, two separate liposomal systems containing DOX (Lip-DOX) and MET (Lip-MET), respectively, were developed and coadministered individually due to stability issues associated with coencapsulation. Both liposomes were prepared using the same lipid composition and under identical experimental conditions, resulting in similar physicochemical properties. While the use of separate liposomes may be seen as a limitation, the literature supports this strategy as a viable alternative when coencapsulation is hindered by physicochemical incompatibilities or stability concerns.^32,33^ Following this approach, subsequent experiments demonstrated promising therapeutic outcomes, reinforcing the potential of liposomal coadministration of MET and DOX for breast cancer treatment.

Previous studies have demonstrated numerous advantages of liposomal formulations, including a significant reduction in toxicity and enhanced uptake by tumor tissue.^34–37^ Therefore, this study aimed to evaluate the potential benefits of the coadministration of liposomes containing DOX and MET. The initial phase of the study involved the preparation and characterization of the liposomes to confirm their suitability for intravenous administration. The Lip-MET and Lip-DOX showed mean diameters around 100–150 nm with a monodisperse distribution. This size range is crucial for consistent drug delivery as it allows the liposomes to remain in the bloodstream, thereby increasing the chances of reaching the tumor site. It is worth noting that liposomes with smaller diameters may present limitations such as low encapsulation efficiency and accelerated renal clearance.^38,39^ In contrast, liposomes with a size greater than 300 nm are more prone to opsonization and activation of the Mononuclear Phagocytic System (MPS), reducing their circulation time and, consequently, accumulation in the tumor site.^40–42^

The encapsulation efficiency of DOX in liposomes was close to 100%, consistent with previous studies carried out by our research group and commonly described in the literature.^21,43–45^ The ammonium sulfate in liposomes facilitates DOX precipitation after entrapment, driven by a concentration gradient. As a result, DOX remains entrapped inside the liposome with high encapsulation efficiency.^45,46,46–50^ For Lip-MET, encapsulation efficiency was around 10%, which is also in agreement with those reported by other authors.^42,51,52^ It is known that low molecular weight and highly hydrophilic molecules, such as MET, often present limited encapsulation efficiency in liposomes. Some studies have reported encapsulation in the order of only 5% for this drug.^51^ It is important to highlight that despite the low encapsulation efficiency of MET, its high aqueous solubility and favorable cost allowed the liposome preparation to start with a concentrated drug solution (50 mg/mL), resulting in MET liposomes with a final drug concentration of approximately 5 mg/mL. The resulting drug load was adequately high for subsequent in vivo studies.

Regarding the release profile, it was seen that the physicochemical properties of MET have a great impact. MET molecules move freely across the dialysis membrane, which would explain the high release of free-MET at all time points compared to the MET liposome formulation.^52^ The release of free-MET was notably fast, as expected. It was observed that 90% of the drug was released within the first 30 min, reaching a plateau after this time point (Figure 2). In contrast, Lip-MET controlled the release of MET, reaching approximately 70% release in 30 min and remaining stable after 60 min. This release profile was comparable to other studies, in which MET liposome made by dipalmitoylphosphatidylcholine and cholesterol reached a release of 40% in 120 min, 60% in 360 min, and continued with the controlled release until 720 min. This formulation was placed in dialysis bags with an external phase composed of ethyl alcohol and saline buffer (20:80) at pH 6.8. The composition of the liposomes, the pH, and the external phase composition could explain the slightly more controlled release than that observed in this study.^53^ In another study, the release profile of the MET liposome showed a more sustained release, with 60% in 360 min, and reaching a constant 80% release until 720 min. As discussed previously, the lipid composition, which provides rigidity to the membrane, and the acceptor phase used in the dialysis could explain a lower drug release;^54^ however, the lipid composition is crucial for fusogenic properties in our study. For DOX-loaded liposomes, the release profile was omitted, as this parameter has already been characterized by our research group, demonstrating a controlled release compared to the free drug. This underscores the formulation’s efficiency in retaining encapsulated DOX, contributing to the overall effectiveness and safety of the treatment.^23,55^

The 4T1 breast tumor is a widely used model for the triple-negative subtype of breast tumor. These tumors are known as aggressive cancers with high progression rates and are often difficult to treat.^29^ This study demonstrated that the combination of DOX and MET, mainly in encapsulated form, positively reduced the progression of the tumor. Studies have shown that the liposomal combination of DOX and MET has emerged as a promising strategy for the treatment of breast cancer, especially in the most aggressive forms, such as the triple-negative subtype represented by the 4T1 model. The coadministration of Lip-DOX and Lip-MET has demonstrated not only a significant reduction in tumor progression, but also greater pharmacological stability and bioavailability of the drugs. Recent studies showed that the encapsulated combination of DOX/MET in liposomes not only potentiated cytotoxicity against tumor cells but also decreased the tumor growth rate compared to free drugs.^15^ In addition, the DOX/MET combination in liposomes was able to bypass multidrug resistance and reduce cell proliferation.^15,56,57^ This synergistic effect can be explained by the ability of MET to modulate the tumor microenvironment and intratumor hypoxia, which potentiates the action of DOX in different tumors.^58,59^ Furthermore, the controlled release provided by the liposomal formulation contributes to a more targeted distribution to the tumor tissue, minimizing systemic side effects.^15,23^ Additionally, hypoglycemia could be raised as a potential concern in Lip-MET protocols. However, in a preliminary study conducted by our group, no significant alterations in glycemia levels were observed in healthy mice treated with our MET regimen (data not shown).

The antitumor efficacy data already mentioned in Section 3.4 corroborate reports on the advantage of the combination of DOX and MET for antitumor efficiency. Figure 3 shows that tumors in the control group have a larger volume when compared to the other treatment groups. Additionally, the tumor volume for the group treated with free-DOX is also significantly higher when compared to the groups treated with Lip-DOX + free MET and Lip-DOX + Lip-MET.^60,61^ Similar results were reported by Li et al. (2019),^15^ who demonstrated the advantage of encapsulating the MET/DOX combination in liposomes to control tumor growth with a cumulative DOX dose of 24 mg/kg. In the present study, the combination of Lip-DOX + Lip-MET achieved a comparable tumor growth inhibition with a lower cumulative dose (20 mg/kg). These findings emphasize the maintained antitumor efficacy and suggest a potential improvement in the treatment’s safety profile. In addition, histopathological analyses were performed to identify necrosis areas in the primary tumor and metastasis in the lung and liver tissues. The sections of the primary tumor (Figure 4) demonstrated morphological characteristics compatible with murine breast carcinoma of 4T1 cells.^19^ The tumors exhibited areas of significant necrosis in animals treated with free DOX, free DOX + free MET, Lip-DOX + free MET, and Lip-DOX + Lip-MET, probably due to the mechanisms of action of drugs in regulating the cell death.^42,62^ In addition, skin ulcers in the tumor area were observed in all fragments for animals receiving Lip-DOX + Lip-MET, an additional signal of cell death.^30^

Liver and lung metastasis are a clinical problem for women with breast cancer. 4T1 breast tumors are characterized by their ability to metastasize to other organs, including the lungs and liver.^29^ Previous studies have shown that DOX and MET, alone or in combination with other drugs, were able to considerably reduce the presence of metastasis foci in organs such as the lung.^64–66^ A significant decrease in the number of metastatic foci was also demonstrated in the group treated with Lip-DOX + Lip-MET compared to the control group. These results highlight the efficiency of combining drugs, proving to be an interesting strategy for inhibiting tumor growth and reducing the incidence of metastases in other organs.^67^ This effect extends to the liver, where the control group showed multifocal metastases with significant inflammatory cell infiltrate. Several studies have demonstrated an inflammatory response correlated with hepatic metastatic foci, as a consequence of the presence of breast cancer cells.^68^ However, in all treatment groups containing free or encapsulated DOX, only focal metastases were observed, with the presence of localized perivascular degeneration. Furthermore, no apparent toxicity was demonstrated in histological sections of the liver and kidneys, indicating that, in addition to significantly reducing the foci of hepatic metastasis, the drug toxicity in these organs was mitigated.

Cardiac toxicity analyses were performed, as it is known that DOX promotes dose-dependent cardiotoxicity. The formation of reactive oxygen species (ROS) is proposed to contribute to DOX-induced cardiotoxicity. DOX-induced ROS production leads to protein, lipid, and DNA damage. It also interferes with mitochondrial functions, decreasing ATP production and compromising cardiac cell viability.^30,31^ Morphologically, cardiomyocyte damage causes hyaline degeneration, characterized by hypertrophic, eosinophilic, and vacuolated cardiac fibers, as observed in this study. However, DOX nanoencapsulation can reduce the incidence and extension of cardiac lesions compared to the free drug.^69^ In this study, as predicted, all animals treated with DOX (free or liposomal) presented areas of hyaline degeneration; however, a decrease in these areas was observed in animals treated with Lip-DOX + free-MET and Lip-DOX + Lip-MET when compared to those treated with free-DOX and free-DOX + free-MET, further supporting the role of DOX encapsulation in cardioprotection.

The ECG analyses of the treated tumor-bearing mice were also performed. Despite the absence of prolongation in classical ECG parameters impacted by DOX treatment, such as QT, QTc, and QRS intervals,^70^ other electrocardiographic parameters, such as arrhythmias, were observed.^71^ The present study revealed an increase in cardiac arrhythmia events in the groups treated with free-DOX, free-DOX + free-MET, and Lip-DOX + free-MET compared to the Lip-DOX + Lip-MET group. This finding highlights that DOX encapsulation, along with its combination with Lip-MET, effectively reduced the incidence of these events. These results align with previous findings suggesting a potential reduction in arrhythmia incidence in animals treated with both encapsulated drugs.^63,72^

## 5 Conclusions

In this study, liposomes carrying DOX (Lip-DOX) and MET (Lip-MET) were developed and characterized. The nanoencapsulation of the drugs allowed an increase in antitumor activity, where Lip-DOX + Lip-MET demonstrated the ability to reduce the rate of tumor growth when compared to the other groups, as well as significantly reducing arrhythmic effects. Furthermore, in animals treated with the combined drugs, there was a significant reduction in liver and lung metastases. Importantly, no significant signs of systemic toxicity were observed for the drug combination in liposomes, encouraging further safety studies. These results highlight the potential of combining MET and DOX as a promising therapeutic strategy for breast cancer treatment.

## List of abbreviations

AMPK: AMP-activated protein kinase
ANOVA: Analysis of variance
ATP: Adenosine triphosphate
CEBIO: Centro de Bioterismo da Universidade Federal de Minas Gerais
CEUA: Comissäo de Ética em Uso de Animais
CHEMS: Cholesteryl hemisuccinate
s.d.: Standard deviation
DLS: Dynamic Light Scattering/Dynamic Light Scattering
DMEM: Modified Eagle Medium
DNA: Deoxyribonucleic Acid/Deoxyribonucleic Acid
DOPE: Dioleoylphosphatidylethanolamine
DOTAP: 1,2-Dioleoyl-3-trimethylammonium-propane
DOX: Doxorubicin
DSPE-PEG2000: Distearoylphosphatidylethanolamine-*N*-(polyethylene glycol)­2000
ECG: Electrocardiogram
EE: Encapsulation Efficiency
FBS: Fetal Bovine Serum
HR: Heart Rate
IC: Tumor Growth Index
Lip-DOX: Doxorubicin Liposome
Lip-MET: Metformin Liposome
LUV: Large Unilamellar vesicles
MET: Metformin
mTOR: Mammalian target of rapamycin (mTOR)
RTV: Relative Tumor Volume
IP: Polydispersity Index
PEG: Polyethylene glycol
MPS: Mononuclear Phagocytic System
UV–vis: Ultraviolet Visible
